# NS1-mediated delay of immune activation contributes to influenza A virulence in ferrets

**Published:** 2011-01-10

**Authors:** I Meunier, V von Messling

**Affiliations:** 1 INRS-Institut Armand-Frappier, University of Quebec, Laval, Quebec H7V 1B7, Canada

## 

Most influenza A viruses cause a mild to moderate disease of the upper respiratory tract with low mortality levels, but certain strains are associated with a severe disease involving the lung and high mortality. Among the proteins known to contribute to virulence, the nonstructural protein NS1 has been identified as the main viral immune interference *in vitro*, and viruses lacking NS1 are highly attenuated in different animal models. To investigate if NS1 proteins of virulent strains are more efficient in interfering with innate immune activation, we generated recombinant USSR/90/77 viruses expressing the NS1 protein of either the attenuated strain PR/8, or the highly pathogenic 1918 “Spanish flu”, all belonging to the H1N1 subtype. While all three NS1 proteins interfered with type I IFN-mediated signaling, assessed by a luciferase reporter gene assay, the 1918 NS1 protein was significantly more efficient. Moreover, when cells were infected with the recombinant viruses, we observed that the presence of PR/8 NS1 was associated with an earlier and overall stronger type I IFN induction. Infection of ferrets with the different viruses revealed that the virus with the 1918 NS1 protein caused a more severe disease with overall higher clinical scores and a higher fever peak. The presence of NS1 from virulent strains correlated with a delay in virus clearance from the upper respiratory tract and spread to the lungs. Moreover, these viruses caused more lung damage with partial loss of the bronchial epithelial layer and alveolar swelling that persisted for up to four days. In contrast, the recombinant virus expressing the PR/8 NS1 protein resulted in little histopathological damage but slightly more inflammation. To assess the impact of the different viruses on immune activation in the upper respiratory tract, IFN and cytokine mRNA induction levels in nasal wash cells were quantified. Consistent with the *in vitro* studies, the virus with the PR/8 NS1 protein induced significantly more IFN-β at days 1 and 3 post-infection, and more IFN-α at day 1 post-infection. In contrast, presence of the more virulent USSR and 1918 NS1 proteins resulted not only in inhibition of early type I IFN induction, but was also associated with a delayed expression of the pro-inflammatory mediators, TNF-α and IP-10. Taken together, these results demonstrate the importance of NS1-mediated immune interference constitutes for influenza A virus virulence.

